# Analysing the Porosity Distribution in Stone Surfaces by Means of Unilateral NMR after Long-Term Outdoor Weathering

**DOI:** 10.3390/ma15134604

**Published:** 2022-06-30

**Authors:** Melanie Groh, Jeanette Orlowsky, Robert Schulte Holthausen

**Affiliations:** 1 Chair of Building Materials, TU Dortmund University, August-Schmidt-Str. 8, 44227 Dortmund, Germany; jeanette.orlowsky@tu-dortmund.de; 2 Ardex GmbH, Friedrich-Ebert-Straße 45, 58453 Witten-Annen, Germany; robert.schulte.holthausen@rwth-aachen.de

**Keywords:** unilateral NMR, natural stone, long-term weathering, porosity distribution, porosity changes

## Abstract

Porosity changes in the near-surface area of sandstones due to long-term weathering can produce deterioration. Therefore, porosity analyses on weathered sandstones are significant for detecting possible influences on the pore structure. Classical methods for determining the porosity and pore size distribution in sandstones can only investigate the entire sample volume. In contrast, in this publication, the porosity was analysed in 0.2 mm steps over a depth of 5 mm by means of single-sided NMR measurements on water-saturated sandstones under vacuum. Evaluations of Obernkirchener and Schleeriether Sandstones that were weathered outdoors in Germany for over 30 years are presented. The results showed that the water content in Vol.-% strongly correlated with the normalised NMR signal. The unweathered sandstones showed a uniform distribution of micro and capillary pores throughout the stone depth. As a result of 30 years of outdoor weathering, changes in the pore structure occurred at the sandstone surface due to weathering down to depths of about 0.6 mm. The porosity of the Schleeriether Sandstone samples, mainly the microporosity, clearly increased in this region. Due to the dominance of capillary pores in the Obernkirchener Sandstone, the changes were not as pronounced, but a shift towards smaller pores in the surface area was observable.

## 1. Introduction

Natural stones are exposed to a variety of external weathering influences over their usage as a building material. Depending on the petrographic and petrophysical properties of the natural stones, environmental factors influence their weathering intensity over time. This can also change the existing stone properties and produce deterioration. In particular, the pore structure of natural stones due to weathering phenomena, especially in the near-surface regions, is of decisive importance with regard to water absorption behaviour and pollutant ingress.

Analysis of the pore structure of mineral building materials is conventionally carried out using mercury porosimetry and additional microscopic transillumination of thin sections [1,2]. Furthermore, investigations using the NMR technique produced successful porosity analyses of concrete samples [3,4]. The study presented in this paper was intended to evaluate the extent to which nuclear magnetic resonance spectroscopy could be used as a non-destructive measuring method to evaluate the depth-dependent detection of the porosity distribution and changes in weathered natural stones. 

## 2. Materials and Methods

From 1985 to 1996, a BMFT (Federal Ministry of Research and Technology, Bonn, Germany) Priority Programme focused on the decay and conservation of natural stones. In this context, several outdoor field studies were conducted to analyse the weathering processes of different stone materials by considering their influencing factors, as well as testing suitable protective agents on the samples. One of these studies was launched by the Zollern Institute (today: Deutsches Bergbau-Museum Bochum) in the year 1986/1987. In this study, several natural sedimentary stones were exposed at different weathering locations in Germany (three sites each in North Rhine-Westphalia and Bavaria) for up to 30 years. The used sample material consisted mainly of sandstones and limestones, which are frequently used as building materials but also have a comparatively high susceptibility to weathering [5,6].

Two representative stone types of the outdoor field study were Obernkirchener (OKS) and Schleeriether Sandstones (SST), which were used for the investigations in this study. The beige-to-yellowish-grey Obernkirchener Sandstone can, for example, be found as a load-bearing building material in sacred buildings, such as the Willibrordi Cathedral in Wesel and partly in the Royal Palace in Amsterdam. Nowadays, it is increasingly used for facade panels. Schleeriether Sandstone is a light-green-to-grey sandstone and is often used in the Bavarian region for solid constructions, such as the Würzburg Residence, but also for structuring elements on facades, sculptures and monuments [7].

Table 1 summarises the petrographic and petrophysical properties of both of these stone types. The microstructure of OKS is characterised by solid grain-to-grain and sutured contacts and kaolinite is included as a pore space filler. Its weathering resistance is considered very good to good. In addition to discolouration, biogenic growth and black crusts, the weathering phenomena of the Obernkirchener are rarely scaling and spalling. The clayey-chloritic matrix of SST contains punctate and elongated grain contacts and the grains are mostly covered with chlorite. Overall, the weathering resistance of Schleeriether Sandstone is classified as moderate to good. Due to outdoor weathering, discolouration, sanding, crumbling and occasional flaking occur in protected areas. After longer exposition times, scaling and salt damage, especially in the base areas, can be observed [2,7].

**Figure 1:**
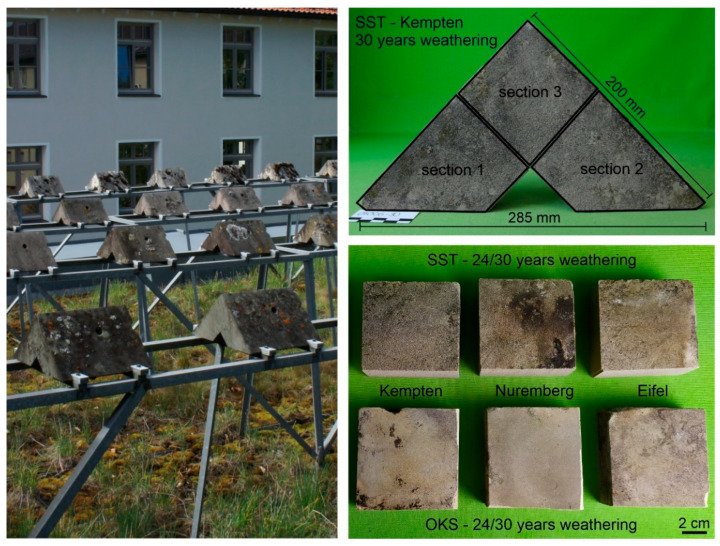
**Left**: exposure site in Kempten, situated on the roof of a tree nursery (residential area). **Top right**: sample preparation after 2 and 24/30 years of exposition. **Bottom right**: weathered sections of Obernkirchener and Schleeriether Sandstones due to long-term exposure.

The test specimens for the field study had a triangular prismatic shape to simulate typical conditions on buildings. A part of the samples was impregnated with organosilicon-based hydrophobing agents before exposition, while the other samples remained untreated [5]. The untreated samples exposed in Eifel, Nuremberg and Kempten were the focus of this study (Table 2). A detailed description of the climatic data and pollutant values at the weathering locations over the last 10 years of exposure can be found in Orlowsky et al. [10]: Overall, Nuremberg had a lower number of hours with relative humidity above 80% than the other two locations. In contrast, the pollutant input was the highest in Nuremberg (urban area) compared with the others. At the test locations, the specimens were placed 1 m above the ground on a metal frame (Figure 1, left). Two years after the exposition, a 3 cm thick slice was cut off each prism and afterwards stored indoors. After 24 resp. 30 years of natural weathering of the remaining prisms, the samples were removed and a second slice was cut off the other side. Additional samples were stored indoors as references. In order to carry out various measurements on the samples in the laboratory [10,11], the slices were cut into several sample sections and used for individual investigations (Figure 1, top right).

The samples considered in this study already showed darkening of the surface and biogenic growth after 2 years of weathering. These damage patterns increased only slightly over the course of the long-term exposure (Figure 1, bottom right). Overall, macroscopic and microscopic examinations of other sample sections of the two stone types showed a considerable change in appearance due to long-term weathering, with the Schleeriether Sandstone showing less color change over the exposition time. The analysed black and covering weathering layers on the surfaces consisted of accumulated particles on existing biofilms (more pronounced growth in terms of height for the Obernkirchener Sandstone samples). By means of XRD investigations, different types of salts (especially sulfates and nitrates) and, to a lesser extent, carbonates were confirmed as present in the surface deposits of the Schleeriether Sandstone samples [1,2].

In order to be able to analyse the possible structural changes in the near-surface area of the samples due to the outdoor weathering, different investigation methods were used. 

### 2.1. Mercury Porosimetry

One of the most common methods for determining the pore volume, pore size distribution and total porosity of natural stones, besides gas adsorption, is mercury porosimetry according to DIN ISO 15901-1 [8]. For this purpose, sample material (pieces with a size of approx. 5 mm × 5 mm) was taken from the area near the surface (in the first 4–5 mm) of sample section 2. The stone fragments were dried at 40 °C until a constant mass was reached. The drying process was carried out as gently as possible in order to prevent temperature-sensitive stone components from being affected and subsequently falsifying the measurement results. After that, they were stored in an exsiccator until they were measured. 

The measuring method using mercury is based on the non-wetting property of this heavy metal (contact angle between 125° and 150°). Under vacuum, mercury is pressed through the pore entrance into the open pore space. In this process, the applied pressure is inversely proportional to the pore inlet radius. Assuming that all pores are cylindrical capillaries, the pore size distribution can be determined using the Washburn equation [12].

Thus, this measuring method detects the pore spaces that are accessible for possible transport processes of liquids or gases. The pore entrance radius, as the connecting point of interconnected pores, is of decisive importance and this value is measured with the mercury porosimetry [13,14]. 

According to Klopfer [15], pores of building materials can be divided into three categories: micropores: <0.1 µm, capillary pores: 0.1 µm ≤ d < 1000 µm and macropores: ≥1000 µm. Micropores, which are responsible for diffusion transport and capillary condensation, can be detected via mercury porosimetry down to a minimum pore diameter of approx. 0.008 µm (according to the manufacturer’s specifications of the measuring device used: Pascal 140–240 Porosimeter from the company Porotec). The relevant capillary pore fraction of these sandstone types, which is important for capillary and water vapour diffusion transport, can be recorded entirely with the dilatometer (CD 6) used for the measurement because pore diameters of up to 100 µm can be detected with it. 

**Figure 2:**
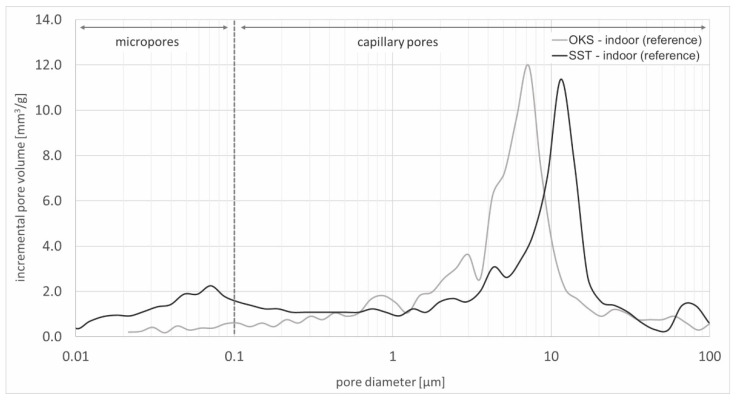
Pore size distribution of the Obernkirchener and Schleeriether Sandstones stored indoors (reference).

Thus, in Figure 2, the pore size distribution of the Schleeriether and Obernkirchener Sandstone samples revealed that both types of stone contained micropores, as well as capillary pores. However, a higher proportion of micropores was present in the Schleeriether Sandstone samples. Furthermore, the average pore diameter was larger than for the Obernkirchener samples.

### 2.2. NMR Measurements

To obtain a non-destructive statement about the water and porosity distribution of the investigated material, measurements were carried out by using a unilateral NMR device (Profile NMR-Mouse^®^, PM 25) [16,17]. A single-sided inhomogeneous stray field, which is set up by four permanent magnets, aligns the atomic nuclei (^1^H) of water inside the sample in the field’s direction. By emitting high-frequency pulses (sent from a coil placed between the permanent magnets) during the measurement, the atomic nuclei are deflected within a measuring volume of roughly 40 mm × 40 mm × 0.2 mm and their response is detected [18]. 

The received response is transmitted to a spectrometer (Kea2) and visualised via data processing software in the form of unitless diagrams. Individual depth-dependent amplitudes (A) and relaxation times (T_2_) can be extracted from the recorded decay curves. Thus, a detected decay curve always consists of several individual exponential functions, which can be assigned to the different types of pores present in the samples. The separation of the individual signals takes place either through a multi-exponential fit or inverse Laplace transformation [4,19]. Further information on the detected number of relaxation components and the method of curve fitting used for the available sample material follows in Section 3. 

In order to determine the most suitable measurement parameters for the available samples, several preliminary tests were carried out. The measurement parameters derived from the preliminary investigations are listed in Table 3. 

The relaxation signals measured with the unilateral NMR device are always relative values without quantitative reference and are subject to measurement-dependent deviations and scattering. Since the quantitative content of water within the samples needed to be determined in this work in order to derive the existing porosity and distribution, pure water was used for the previous standardisation [3,18,20]. The deviations of the first echoes were corrected (correction factor 1. echo = 0.75) and the resulting corrected amplitudes were normalised to the amplitude of pure water (A_w_ = 2.26, T_2w_ = 104 ms).

**Figure 3:**
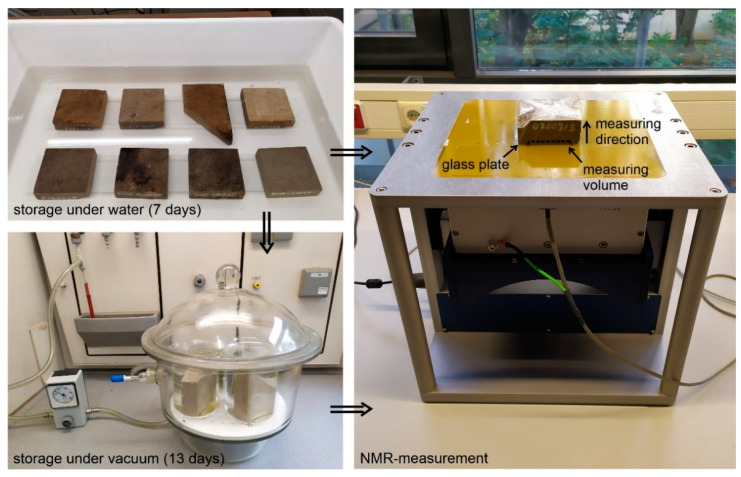
**Top left**: storage of the samples under water at atmospheric pressure (1 bar) for 7 days. **Bottom left**: then, the samples were stored at negative pressure (0.05 bar) for 13 days. **Right**: measuring the relaxation times after each storage period with the unilateral NMR device.

In order to obtain sufficiently precise information about the penetration behaviour of water into the mineral surface structure of the investigated natural stones, the test specimens were prepared accordingly. In order to identify different saturation levels, the dry samples (stored in a room climate) were first stored under water at atmospheric pressure (1 bar) for 7 days according to DIN EN 13755 [21] (Figure 3, top left). To avoid surface effects that prevent the further filling of pores in the sample depth, as described in Bortolotti et al. [22], the specimens were subsequently exposed to a negative pressure of 0.05 bar for a period of 13 days (Figure 3, bottom left). For each storage period, a sample was taken out of the water bath and prepared for measuring. First, the excess surface water was blotted off and a 1 mm thick glass plate was placed on the weathered surface. Then, the sample was wrapped in a one-layer vapour-tight polyethylene film and placed with the weathered side downwards on the NMR device (Figure 3, right). The glass plate prevented direct contact between the film and the sample surface, which separated the barely distinguishable NMR signals from the plastic film and material and allowed for the sample surface to be detected more accurately [4].

## 3. Results

**Figure 4:**
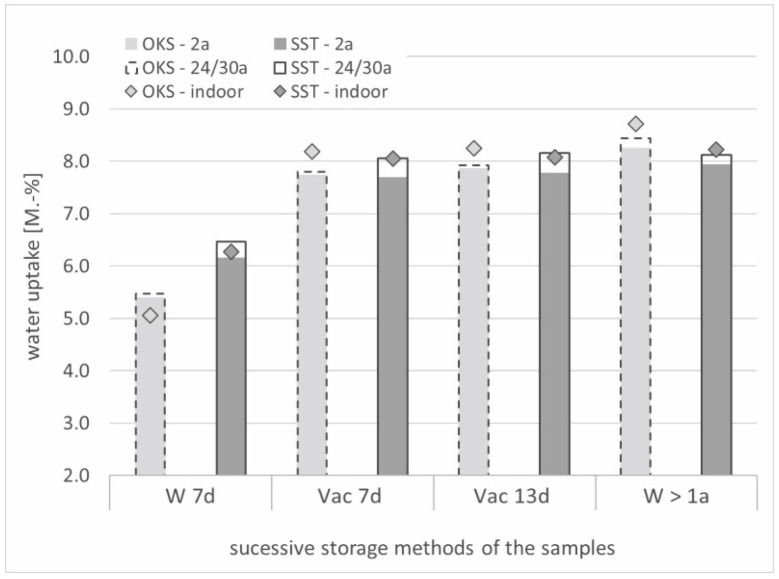
Water ingress of Obernkirchener and Schleeriether Sandstone samples over successive storage methods. Mean values of the different outdoor exposures (see Table 2).

To evaluate the amount of water absorbed by each sample during the different storage conditions, their weight was determined gravimetrically before each NMR measurement (Figure 4). After the analysis of the 13-day vacuum-saturated samples, they were put back into the water and stored there for more than 1 year (on average, a total of 378 additional days) for a long-term study. 

Comparing the gravimetrically determined water uptake of the two stone types after the first 7 days of water storage (1 bar), the exposed Schleeriether Sandstone (SST) samples recorded higher values than the Obernkirchener Sandstone (OKS) samples. The application of negative pressure (0.05 bar) during underwater storage caused a stronger water ingress in the Obernkirchener samples than in the Schleeriether samples such that a similar level was reached after 13 days. In addition, the Obernkirchener samples showed a further increase as a result of long-term exposure to water. In comparison, the water absorption of the unweathered (indoor) samples of both stone types showed related percentage increases. The exposition time of 24/30 years had no influence on the water uptake of the Obernkirchener Sandstones. Only the long-term-exposed Schleeriether samples had an increased uptake compared with the samples exposed only for 2 years.

**Figure 5:**
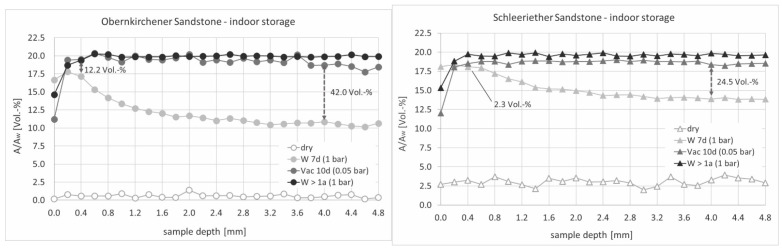
Depth profiles at different saturation levels for unweathered Obernkirchener and Schleeriether Sandstones.

The results of the destructive gravimetric method showing incomplete saturation after only water saturation were further confirmed via depth profiles of the unilateral NMR measurements (based on the measured relaxation curves corrected with the given parameters and normalised to the amplitude of pure water), which additionally allowed for depth-dependent statements to be made about the water distribution within the samples (Figure 5). For example, the high amplitude values in the first 400 µm of the 7-day water-stored Obernkirchener reference sample indicated a water enrichment in this near-surface area, whereas lower values were present at a greater depth (4000 µm). In the case of the Schleeriether Sandstone sample, more water had penetrated the sample depth during the saturation with 1 bar, which could be recognised by the higher amplitude values there. Thus, the Schleeriether Sandstone showed a less pronounced surface effect. As a result of the saturation under vacuum, the surface effects disappeared, which led to an equalisation of the amplitude values in the sample. In the deeper layers, higher signal amplitudes were achieved than with standard pressure (1 bar), which reached a constant level throughout the depth.

**Figure 6:**
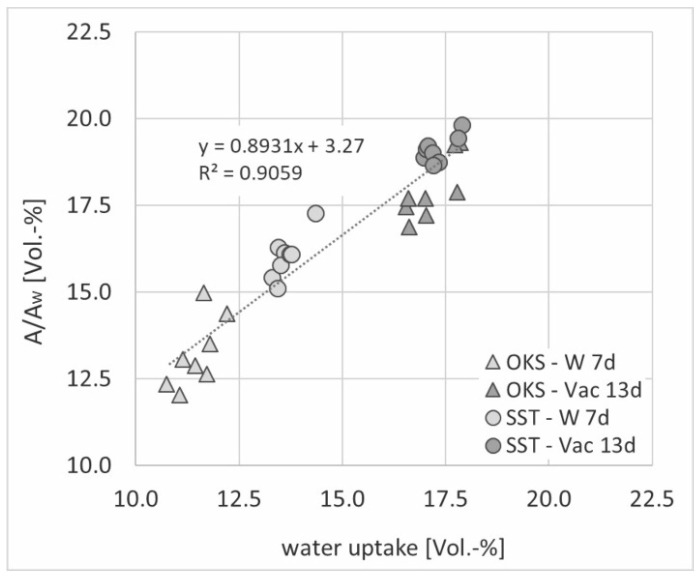
Correlation of gravimetric water uptake and measured NMR signal normalised to A_w_.

Based on the gravimetrically determined water absorption values, the porosity of the individual samples could be calculated according to DIN EN 772-4 [23]. Thus, the generated values for the samples saturated using negative pressure (Vac 13d) reflected the open porosity of each sample. The comparison of the gravimetric water absorption values with the measured and normalised NMR signals (averaged over the entire depth of the samples considered) at the different saturation levels showed a positive linear correlation with minimal scattering due to the natural internal stone heterogeneity (Figure 6). Similar results were generated for both stone types for saturation under atmospheric pressure (W 7d) and under vacuum (Vac 13d). Overall, a shift due to slightly higher normalised NMR amplitude values, especially for the Schleeriether Sandstone samples, could be detected. This may have resulted from detected amplitude signals due to residual stone moisture in the dry state (storage at room climate) of the samples (for SST-dry: A/A_w_ ≈ 2.5 Vol.-%, for OKS-dry: A/A_w_ ≈ 0.9 Vol.-%; see Figure 5—dry samples). When the samples were exposed to water for more than a year, comparatively higher NMR signals were obtained for both stone types, with almost constant gravimetric water absorption values. A possible influence on the results due to different degrees of weathering of the samples was not detectable. Overall, this showed once more that this NMR measuring method is well suited for determining the porosity of different natural stone types [24].

In order to obtain further information from the measured exponential relaxation curves about the existing porosity, as well as different pore sizes in individual depths of the samples, a curve fitting was performed by means of a multi-exponential fit [4]. Based on the two types of pores present in the investigated sample material listed in Section 2, which were detected using mercury porosimetry, two relaxation components were assumed, and thus, a bi-exponential fit was used for the evaluation. 

The individual T_2_ relaxation times generated from the decay curves depend on the properties of the pore surfaces and provide information about the pore sizes [25]. Thus, the component with the shorter T_2_ relaxation time (T_2-1_) indicates the detection of pores with small radii, in this case, micropores. The corresponding amplitude values (A1) provide information about the present quantity of micropores at the depth considered in each case. The capillary pores are detected via a longer T_2_ relaxation time (T_2-2_) and in accordance with the A2 amplitude values.

**Figure 7:**
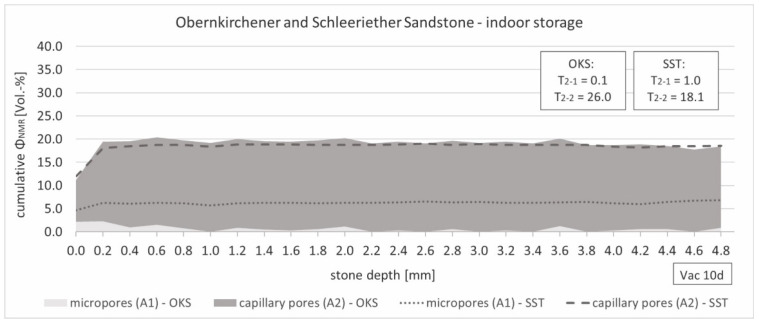
Fitted and stacked relaxation depth profiles of unweathered Obernkirchener and Schleeriether Sandstone samples saturated under vacuum (0.05 bar).

As Figure 7 shows, the unweathered sandstone samples of both stone types saturated under vacuum showed a constant porosity distribution over the entire measuring depth of 4800 µm. Despite the approximately equal total porosity of both types of stone, quantitative differences in the present types of pores could be determined. One-third of an average Schleeriether Sandstone sample consisted of micropores, while in the Obernkirchener samples, the capillary pore fraction dominated the pore structure. This correlated with the pore size distribution of the two stone types measured using mercury porosimetry (see Figure 2).

**Figure 8:**
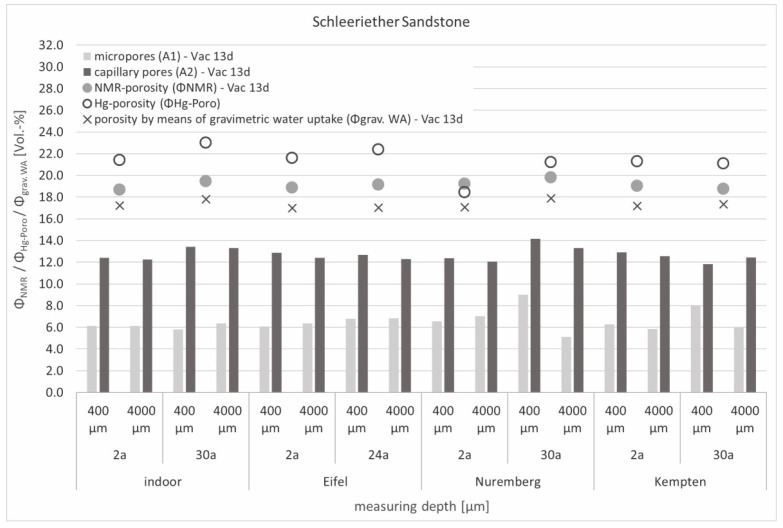
Distribution of micro (A1) and capillary (A2) pores within the Schleeriether Sandstone samples that were measured in the near-surface area (400 µm) and the sample depth (4000 µm), and presentation of the resulting NMR-porosity (Φ_NMR_) (averaged over the entire depth of the samples) in comparison with the detected porosity measured using mercury porosimetry (Φ_Hg-Poro_) and gravimetric water uptake (Φ_grav_. _WA_).

The determination of the porosity using NMR showed lower values than those measured using mercury porosimetry for almost all Schleeriether Sandstone samples (except the samples exposed in Nuremberg) (Figure 8). When the samples were stored in water for more than one year, the NMR values became more and more similar to those obtained with mercury porosimetry. However, since the correlation coefficient regarding water uptake decreased as a result of long-term saturation and such saturation periods are unusual, even for laboratory conditions, the saturation of the samples by means of 0.05 bar negative pressure was still taken as the relevant condition for the detection of porosity. The determination of the porosity by means of gravimetric water uptake showed somewhat lower values for all samples in comparison to the porosities determined using NMR, which correlated with the results presented in Figure 6. Based on the comparison of the pore types present, an increase in micropores on the surface could be observed, especially for the long-term weathered samples exposed in Nuremberg and Kempten. Despite the existing scatter of the measured values due to natural internal stone heterogeneities (for example, indoor storage over 2 and 30 years), similar distributions were found for all samples exposed for 2 years.

**Figure 9:**
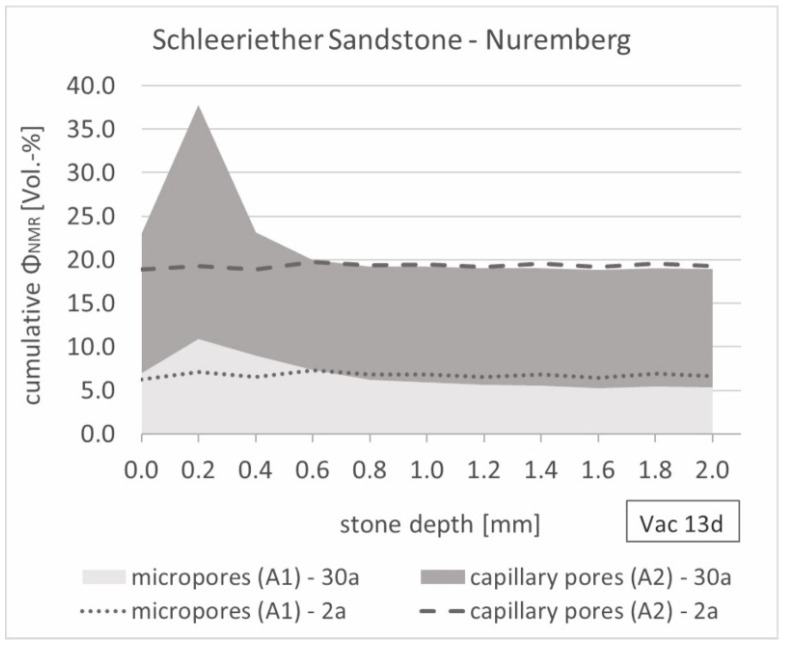
Fitted and stacked relaxation depth profile of the 2- and 30-year weathered Schleeriether Sandstone samples (exposure site: Nuremberg) saturated under vacuum (0.05 bar).

A similar curve progression after 2 years of exposure was also detected for the Nuremberg samples when the distribution was plotted over the measured sample depth (Figure 9). However, after long-term outdoor weathering, an increase in the micro and capillary porosities could be observed in the first 600 µm. Thus, the NMR-porosity at a sample depth of 200 µm increased from 19 Vol.-% (2 years) to 38 Vol.-% (30 years) due to superficial weathering phenomena. The sample material weathered in Kempten showed a similar, but slightly lower, porosity change at the surface.

**Figure 10:**
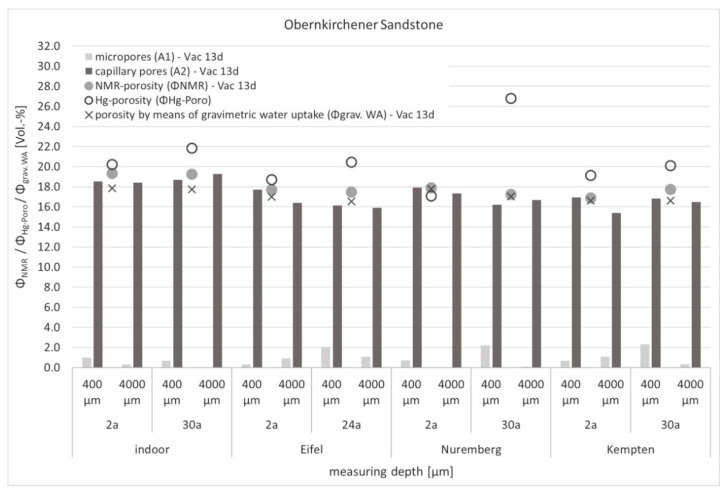
Distribution of micro (A1) and capillary (A2) pores within the Obernkirchener Sandstone samples measured in the near-surface area (400 µm) and the sample depth (4000 µm), and presentation of the resulting NMR-porosity (Φ_NMR_) (averaged over the entire depth of the samples) in comparison with the detected porosity measured using mercury porosimetry (Φ_Hg-Poro_) and gravimetric water uptake (Φ_grav_. _WA_).

**Figure 11:**
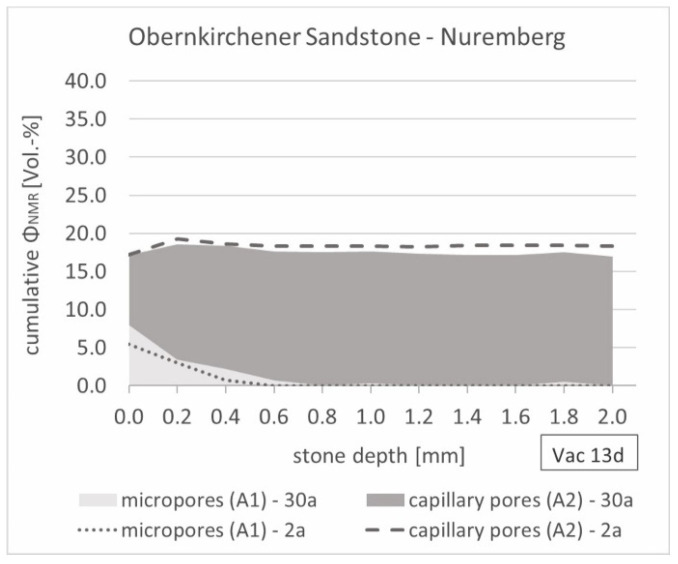
Fitted and stacked relaxation depth profile of the 2- and 30-year weathered Obernkirchener Sandstone samples (exposure site: Nuremberg) saturated under vacuum (0.05 bar).

Comparing the NMR-porosity of the Obernkirchener samples with the results using mercury porosimetry, lower values for almost all samples were detected again, while the porosities, determined using water uptake, showed similar results to the measured NMR-porosities (Figure 10). Overall, the distribution of the two pore types detected showed mainly capillary pores in the Obernkirchener Sandstone samples, which had already been observed in the indoor samples. In particular, within the sample depth, no microporosity was found. Furthermore, a larger scattering of porosities of the individual samples was noticed. Nevertheless, a slight shift towards smaller pore sizes was seen on the surface (400 µm) of the exposed samples as a result of outdoor weathering, which increased somewhat over the long-term exposure (Figure 11).

## 4. Comparison and Discussion

In this study, unilateral nuclear-magnetic resonance was used to gain non-destructive information about the pore size distribution in the near-surface regions of long-term-weathered natural stones. To classify the results and to compare them with conventional destructive measuring methods, the corrected and normalised NMR signals were correlated with the gravimetrically determined water absorption. The linear positive correlation coefficient reconfirmed the results of previous studies on mineral building materials, showing that the NMR-porosity (Φ_NMR_) is a direct measure of the waterfilled porosity of the investigated sample [20]. An influence of the correlation coefficient towards higher NMR signals may result from detected amplitude signals due to residual stone moisture in the dry state of the samples not accounted for in the gravimetric testing.

Due to the required sampling down to depths of about 5 mm for mercury porosimetry, this method cannot be used for a depth-resolved observation of changes in porosity due to weathering. However, these measurements can be made with the NMR method presented here, for which a bi-exponential fit for elevation was used. In order to make a suitable assumption about the number of components present within the samples, it is necessary to consider in advance which samples are involved and which types of pores are to be expected. The examined stone types showed a different distribution of the two types of pores present, which had an influence on the analyses. While the Schleeriether Sandstone had both micro and capillary pores, the capillary pores dominated in the Obernkirchener sandstone. The evaluation of the pore fractions over a stone depth of 5 mm in 0.2 mm steps showed no change in porosity over the depth of the Schleeriether Sandstone in the unweathered state. Outdoor weathering over 30 years in southern Germany caused an increase in porosity at the stone surface down to about a 0.6 mm stone depth, especially for the micropores. However, in the case of Obernkirchener Sandstone, a change in porosity at the stone surface as a result of 30 years of outdoor weathering could not be clearly determined; the capillary porosity tended to decrease at the surface after long-term weathering, while the microporosity increased up to 2 Vol.-%. This could indicate possible densification of the pore spaces at the surface due to covering weathering layers and a reduction in superficially accessible kaolinitic pore space fillings, which was determined on the basis of polarisation microscopy images of other long-term exposed Obernkirchener samples [2]. Further investigations have to be carried out in this regard.

Due to the much larger measurement volume and the possibility of measuring a wide range of different pore radii and sample depths, the results of the NMR measuring method are subject to less scatter than those determined by means of mercury porosimetry.

## 5. Conclusions and Outlook

Overall, the non-destructive measuring method used showed good suitability for the detection of the porosity at individual sample depths. Changes in the pore structure could be determined, especially when comparing different exposure durations. The results presented, which were determined on sandstones that were water-saturated under vacuum, can be summarised in the following key points:

- The Obernkirchener and Schleeriether Sandstones showed a uniform water content over the whole stone depth (measured in 0.2 mm steps) due to saturation under negative pressure (0.05 bar).
- The gravimetrically determined water absorption (in Vol.-%) correlated with the measured NMR signal normalised to A_w_ (in Vol.-%).
- Using a bi-exponential fit of the T_2_ decay curve, the micro and capillary pores that were present in the two sandstones could be distinguished.
- The unweathered sandstones showed no change in porosity distribution over the sample depth, measured in 0.2 mm steps to a 4.8 mm depth. While the Schleeriether Sandstone had 6 Vol.-% micropores and 12 Vol.-% capillary pores, the Obernkirchener Sandstone had only 1 Vol.-% micropores and around 18 Vol.-% capillary pores.
- As a result of 30 years of outdoor weathering in southern Germany, the proportion of pores of the Schleeriether stone surface (the first 0.6 mm) and the permeability of water increased.
- As a result of 30 years of outdoor weathering, the change in the pore structure of the Obernkirchener Sandstone was not as well observable. The proportion of capillary pores on the surface of the stone samples tended to decrease, while the micropores at a stone depth of 0.4 mm increased to about 2 Vol.-% for all exposure sites.

In the next steps, the results will be verified by further comparative investigations, such as microscopic examinations. Furthermore, this method for porosity analysis should also be adapted for hydrophobic long-term-weathered natural stone samples. This could provide a good basis for establishing single-sided NMR as a non-destructive measuring system to carry out in situ porosity measurements of building structures.

## Figures and Tables

**Table 1:** Petrographic and petrophysical properties of Obernkirchener and Schleeriether Sandstones. The total porosity and average pore radius were measured using mercury porosimetry according to DIN ISO 15901-1 [8]. The water absorption coefficient was determined using capillary water absorption according to DIN EN 15801 [9].

Stone Type	Obernkirchener Sandstone (OKS)	Schleeriether Sandstone (SST)
Characterisation [7]	Fine-grained, well-sorted quartzitic sandstone	Fine-grained, moderately sorted sandstone
Mineral content [1,2]	Quartz 85%, rock fragments 10%, muscovite 5%	Quartz 65%, rock fragments 20%, muscovite 10%, feldspar 5%
Matrix [7]	Quartzitic, kaolinitic	Clayey-chloritic
Weathering damage [7]	Black crusts, spalling	Scaling, sanding, salt damage
Total porosity (%)	20	21
Average pore radius (µm)	3.4	6.0
Water absorption coefficient (kg/(m² × h^0.5^))	1.38	2.17

**Table 2:** Used stone types and their exposition.

Stone Type	Exposure Site	Exposition Time
Obernkirchener Sandstone (OKS)	Indoors	-
	Eifel	2 and 24 years
	Nuremberg	2 and 30 years
	Kempten	2 and 30 years
Schleeriether Sandstone (SST)	Indoors	-
	Eifel	2 and 24 years
	Nuremberg	2 and 30 years
	Kempten	2 and 30 years

**Table 3:** Measurement parameters and adjusted values.

Measured Parameter		Adjusted Value	
		OKS	SST
Resolution	µm	200	200
Repetition time	ms	850	850
Pulse length	µs	16	16
Echo time	µs	80	80
Number of echoes	-	100	100
Number of scans	-	200	200
Measuring depth from sample surface	µm	4800	4800
